# Temporal-spatial dynamic functional connectivity analysis in schizophrenia classification

**DOI:** 10.3389/fnins.2022.965937

**Published:** 2022-08-17

**Authors:** Cong Pan, Haifei Yu, Xuan Fei, Xingjuan Zheng, Renping Yu

**Affiliations:** ^1^Henan Key Laboratory of Brain Science and Brain-Computer Interface Technology, School of Electrical Engineering, Zhengzhou University, Zhengzhou, China; ^2^Aviation Maintenance NCO Academy, Air Force Engineering University, Xinyang, China; ^3^School of Artificial Intelligence and Big Data, Henan University of Technology, Zhengzhou, China; ^4^Gaoyou Hospital Affiliated to Soochow University, Gaoyou People’s Hospital, Gaoyou, China

**Keywords:** functional magnetic resonance imaging, schizophrenia classification, dynamic functional connectivity networks, high-order functional connectivity network, temporal variability

## Abstract

With the development of resting-state functional magnetic resonance imaging (rs-fMRI) technology, the functional connectivity network (FCN) which reflects the statistical similarity of temporal activity between brain regions has shown promising results for the identification of neuropsychiatric disorders. Alteration in FCN is believed to have the potential to locate biomarkers for classifying or predicting schizophrenia (SZ) from healthy control. However, the traditional FCN analysis with stationary assumption, i.e., static functional connectivity network (SFCN) at the time only measures the simple functional connectivity among brain regions, ignoring the dynamic changes of functional connectivity and the high-order dynamic interactions. In this article, the dynamic functional connectivity network (DFCN) is constructed to delineate the characteristic of connectivity variation across time. A high-order functional connectivity network (HFCN) designed based on DFCN, could characterize more complex spatial interactions across multiple brain regions with the potential to reflect complex functional segregation and integration. Specifically, the temporal variability and the high-order network topology features, which characterize the brain FCNs from region and connectivity aspects, are extracted from DFCN and HFCN, respectively. Experiment results on SZ identification prove that our method is more effective (i.e., obtaining a significantly higher classification accuracy, 81.82%) than other competing methods. *Post hoc* inspection of the informative features in the individualized classification task further could serve as the potential biomarkers for identifying associated aberrant connectivity in SZ.

## Introduction

Schizophrenia (SZ) is a serious chronic mental disorder (Insel, 2010) that affects the brain, with the symptoms typically including hallucinations, delusions, emotional disturbances and confusion in language and behavior (Galderisi et al., 2018). Due to the complexity of the brain, the heterogeneity of disease, and the overlapping symptoms between different psychiatric disorders, clinical diagnosis is still difficult (Jablensky, 2022). Despite decades of research, the pathological mechanism of SZ is still not well understood. Research suggests that SZ may have differences in the connectivity of brain regions (Lynall et al., 2010; Lin et al., 2021).

Benefiting from the development of neuroimaging and computer technology, resting-state functional magnetic resonance images (rs-fMRI), measuring the low-frequency fluctuations in the blood-oxygen-level-dependent (BOLD) signals, have been proved to capture spontaneous neural activity of the brain that reflects functional organization (Barkhof et al., 2014; Zhang et al., 2019). Graph theory-based brain networks can effectively represent the coordinated interactions of neural activities between different ROIs/nodes and reflect the functional connectivity (FC; edges between nodes) in the complex brain (Vecchio et al., 2017; Sporns, 2022). A series of functional brain network construction methods based on BOLD signals such as correlation-based methods (Sporns, 2011) and sparse-learning-based methods (Yu et al., 2017; Gao et al., 2020) have been widely applied in brain disease diagnosis. For instance, Li et al. (2017) remodeled the functional brain network for autism spectrum disorder diagnosis based on Pearson’s correlation [PC; the most commonly used correlation-based method (Smith et al., 2011)] using sparsity and scale-free prior, respectively. Yu et al. (2019) constructed a weighted graph regularized sparse brain network for mild cognitive impairment diagnosis. However, these methods assumed the temporal stationarity of functional brain network (aka, static functional connectivity network, SFCN) across the duration of the scan, which ignore the rich dynamic information during scan. The dynamic interactions of ROIs may be critical for diagnosing brain disorders.

Researchers suggest that through dynamic reconfiguration of the brain, its various parts can adaptively coordinate and integrate in response to rapidly changing stimuli (Cocchi et al., 2013; Kupis et al., 2021). To capture dynamic interactions between ROIs, dynamic FCN (DFCN), constructed with the sliding window method (Hutchison et al., 2013), could consider the changing characteristics of the brain and simulate these changes by measuring the correlation of brain regions in a short period (Damaraju et al., 2014). For instance, Wee et al. (2016) used the sliding window method and PC to construct a DFCN for the identification of early mild cognitive impairment. Zhang J. et al. (2016) proposed a DFCN-based temporal variability measure to discover differences in the temporal variability of brain networks in subjects with different diseases through the diagnosis of multiple brain diseases. DFCN can simply and effectively represent the dynamic changes in the interaction patterns between ROIs, but cannot represent the complex interaction patterns between multiple brain regions in a deeper way. Neurological findings have demonstrated that a brain region predominantly interacts directly with multiple ROIs in neurological processes (Huang et al., 2010).

High-order functional connectivity network (HFCN) has great potential for mining complex spatial interactions across multiple brain regions. Many studies have proposed different HFCN construction methods. For instance, through extracting the FC series from the DFCN, Chen et al. (2016) proposed to cluster them and constructed the HFCN based on the pairwise correlation of averaged FC series. Zhao et al. (2018) proposed a multi-level HFCN construction method based on SFCN. A multi-level high-order network is obtained through the “correlation’s correlation” strategy, and then features are extracted from each level of the network for autism classification. Li Y. et al. (2020) proposed an ultra-least squares group constrained ultra-orthogonal least squares regression algorithm to construct low-order and high-order brain function networks, and successfully realized the prediction of mild cognitive impairment. High-order networks can provide additional spatial information for disease identification by characterizing complex brain region interactions. However, most traditional functional brain network analysis focus on DFCN or HFCN, which may ignore the complementarity that exists between them.

In this article, we propose a temporal-spatial dynamic functional connectivity method for the diagnosis of SZ. Specifically, the sliding window method is used to construct the DFCN of each subject, and the HFCN is constructed based on DFCN, and then two different features are extracted from these two networks respectively. The features extracted from DFCN reflect the relationship of brain functional network connectivity over time, which called temporal variability. The features extracted from HFCN represent the functional interaction between different modules in the brain network to a certain extent, reflecting the modularity of the brain network, which called spatial variability. Through fusing these two kinds of features that characterize the brain FCNs from region and connectivity aspects, we utilize Least Absolute Shrinkage and Selection Operator (Lasso) (Tibshirani, 1996) for feature selection and build a linear classifier for identification of SZ and HC.

## Materials and methods

### Materials

#### Data acquisition

The rs-fMRI data comes from the Centers of Biomedical Research Excellence (COBRE^1^), which contains 57 SZ subjects and 64 HC subjects. The detailed information of the dataset is shown in Table 1. According to the fourth edition of Diagnostic and Statistical Manual of Mental Disorders (DSM-IV) criteria, the diagnosis of SZ is identified by psychiatrist and symptom is assessed using the Positive and Negative Syndrome Scale (PANSS). All subjects are screened and excluded if (1) history of DSM-IV disorders; (2) history of significant head trauma; and (3) history of substance abuse. The single-shot full k-space echo-planar imaging (EPI) with ramp sampling correction using the inter-commissural line (AC-PC) as a reference is used to obtain rest data, where repetition time (TR)/time-of-echo (TE) = 2,000/30 ms, in-plane voxel = 64 × 64, 32 slices, voxel size = 3 mm × 3 mm × 4 mm, field of view (FOV) = 256 mm × 256 mm and number of volumes = 150.

**TABLE 1:** Demographic information of the participants from COBRE dataset.

	SZ	HC	*P*-value
Numbers	57	64	
Gender (male/female)	48/9	45/19	0.072
Ages	36.684 ± 13.620	35.313 ± 11.804	0.554
Handedness (L/R/both)	8/48/1	1/62/1	**0.022**

The ages are denoted as mean ± SD. L/R/both: left/right/(both left and right). P-value < 0.05 is marked in bold.

#### Data preprocessing

The first 10 volumes are discarded to allow for scanner stabilization and the subjects’ adaptation to environment. The remain volumes are preprocessed using DPABI toolbox (Yan et al., 2016). The processing flow includes slice timing correction, realignment, spatial normalization [a standard Montreal Neurological Institute (MNI) template, resampled to 3 mm × 3 mm × 3 mm], and spatial smoothing using an 8 mm FWHM Gaussian kernel. The band-pass filter (0.01–0.08 Hz) is used to reduce low frequency drift and high frequency physiological noise. The automated anatomical labeling atlas (AAL2) (Tzourio-Mazoyer et al., 2002) is used to parcellate the brain into 120 ROIs. After data preprocessing, the mean time series are extracted from each ROI, and the time point signal of each ROI is normalized.

### Construction of low-order functional connectivity network

#### Construction of static functional connectivity network

Pearson’s correlation measures the functional connectivity of ROIs by calculating the correlation coefficient between the average rs-fMRI time series of ROIs, defines as


(1) $$W_{i\,j}=\frac{{\left(x_{i}-{\bar{x}}_{i}\right)}^{T}\,\left(x_{j}-{\bar{x}}_{j}\right)}{\sqrt{{\left(x_{i}-{\bar{x}}_{i}\right)}^{T}\,\left(x_{i}-{\bar{x}}_{i}\right)}\,\sqrt{{\left(x_{j}-{\bar{x}}_{j}\right)}^{T}\,\left(x_{j}-{\bar{x}}_{j}\right)}}$$

where *x*_*i*_ = [*x*_1*i*_,*x*_2*i*_,⋯,*x*_*Mi*_] ∈ ℝ^*M*^ and *x*_j_ = [*x*_1*j*_,*x*_2*j*_,⋯, *x*_*Mj*_] ∈ ℝ*^M^* denote the average rs-fMRI time series from the *i*-th and *j*-th ROIs respectively. *M* = 140 is the length of the time series. The ${\bar{x}}_{i}$ and ${\bar{x}}_{j}$ are the mean value of *x*_*i*_ and *x*_*j*_ respectively, and *W*_*ij*_ reflects the correlation between the *i*-th and *j*-th ROIs. The whole brain PC matrix is **W** = **X**^*T*^**X** ∈ ℝ^*N*×*N*^, where **X** = [*x*_1_,*x*_2_,⋯,*x*_*N*_] ∈ ℝ^*M*×*N*^ denotes the whole-brain BOLD signals, and *N* = 120 denotes the number of ROIs. **W** characterizes the static correlation of a pair of ROIs throughout the scan time, which ignores dynamic interaction information in the rs-fMRI signal.

#### Construction of dynamic functional connectivity network

**FIGURE 1:**
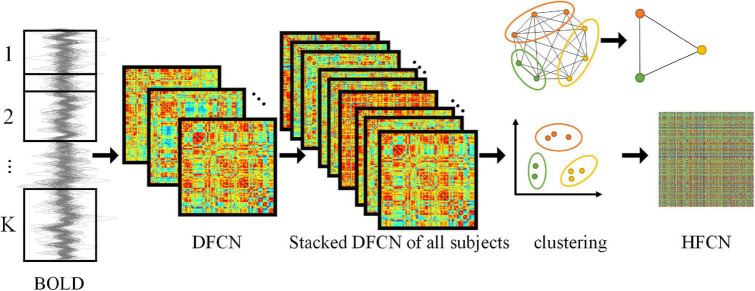
Framework for construction of DFCN and HFCN. The DFCN is first constructed by computing the PC coefficient under *k* different sub-windows based on the BOLD signal. Then the DFCNs of all subjects are stacked to extract FC time series and clustered. Finally, the HFCN is obtained by calculating the PC of different FC sequence clusters. The figure in the upper right illustrates the dimension reduction of higher-order networks through clustering.

To capture the dynamic nature of neural activity, the entire BLOD time series is partitioned into multiple segments of overlapping subseries to construct the sub-networks using a sliding window method. As shown in Figure 1, the number of subseries *K* is


(2) $$K=\left\lceil \frac{M-L}{\Delta }\right\rceil +1$$

where ⌈⌉ denotes the ceiling function, and Δ denotes the sliding step. *L* represents the length of the window. It is worth noting that a smaller *L* can capture short-term inter-ROIs dynamic interactions, but is also more susceptible to noise. Correspondingly, a larger *L* is not conducive to the detection of dynamic interaction information between brain regions. *L* = {20, 30, 40, 50, 60, 70} will be automatically searched in subsequent experiments. The step size is uniformly set to 1, since a smaller Δ can obtain more subsequences to make the results more continuous.

Based on the above, suppose that *x*_*i*_(*k*) = [*x*_1*i*_(*k*), *x*_2*i*_(*k*),⋯,*x*_*Li*_(*k*)] ∈ ℝ*^L^*(*k* = 1,2,⋯,*K*) denotes the average region time series of *i*-th brain region under the *k*-th sub-time window, the PC coefficient of *i*-th and *j*-th ROIs are *W*_*ij*_(*k*). Therefore, the sub-network under the *k*-th sub-time window is defined as


(3) $$C_{D\,L}\,\left(k\right)=\left[\begin{array}{c} W_{11}\,\left(k\right)\,W_{12}\,\left(k\right)\,\cdots \,W_{1\,N}\,\left(k\right)\\ W_{21}\,\left(k\right)\,W_{22}\,\left(k\right)\,\cdots \,\,W_{2\,N}\,\left(k\right)\\ \vdots \;\vdots \;\ddots \;\vdots \\ W_{N\,1}\,\left(k\right)\,W_{N\,2}\,\left(k\right)\,\,\cdots \,\,W_{N\,N}\,\left(k\right) \end{array}\right]$$

where *C*_*DL*_(*k*) represents the low-order DFCN of the subjects under the *k*-th sub-time window. In essence, DFCN reflect the temporal variability of functional connections between ROIs.

### Construction of high-order functional connectivity network

To capture high-order functional interactions across ROIs, the “correlation’s correlation” principle is adopted to obtain HFCN (Chen et al., 2016; Zhang H. et al., 2016). Specifically, the FC series $h_{i\,j}^{s}=\left[W_{i\,j}^{s}\,\left(1\right),W_{i\,j}^{s}\,\left(2\right),\cdots ,W_{i\,j}^{s}\,\left(K\right)\right]\in {\mathbb{R}}^{K}$ between *i*-th and *j*-th ROIs for the *s*-th subject can be obtained from *C*_*DL*_(*k*)(see Eq. 3). $h_{i\,j}^{s}$ reflects the short time-dependent series of the correlation between *i*-th and *j*-th ROIs. Then the high-order connectivity for the *s*-th subject can be computed as $H_{i\,j,p\,q}^{s}=c\,o\,r\,r\,\left(h_{i\,j}^{s},h_{p\,q}^{s}\right)$for each pair of FC series h${}_{i\,j}^{s}$ and $h_{p\,q}^{s}$, where *corr* denotes PC coefficient. Thus, *H* can extract interaction information from up to four ROIs, whereas the correlation in Eq. 3 involves only two ROIs. This suggests that high-order connectivity can characterize more complex interactions.

However, since the number of FC series is *N* × (*N −* 1) / 2, the dimension of the high-order connectivity network grows exponentially with *N*^2^, which will cause a large amount of computation complexity and the poor generalization performance. Therefore, the mean clustering algorithm (Ward, 1963) is employed to group the FC series into different clusters. Specifically, the DFCN of all *S* subjects are stacked together in the following


(4) $$\left\{C_{D\,L}^{1},C_{D\,L}^{2},\cdots ,C_{D\,L}^{S}\right\}\in {\mathbb{R}}^{N\times N\times K\,S}$$

where $C_{D\,L}^{s}=\left\{C_{D\,L}^{s}\,\left(1\right),C_{D\,L}^{s}\,\left(2\right),\cdots ,C_{D\,L}^{s}\,\left(K\right)\right\}\in {\mathbb{R}}^{N\times N\times K}$ denotes the stacked sequence of all *K* sub-networks along the time for the *s*-th subject. Then FC of *i*-th and *j*-th ROIs is denoted by $h_{i\,j}=\left[h_{i\,j}^{1},h_{i\,j}^{2},\cdots ,h_{i\,j}^{S}\right]\in {\mathbb{R}}^{K\,S}$ for all *S* subjects. After that, the *N* × *N* FC series in the stacked network are clustered into *U* clusters, and the corresponding average FC series of *U* clusters are calculated respectively as follows


(5) $${\bar{h}}_{u}=\frac{\sum_{\left(i,j\right)\in \Omega_{u}}h_{i\,j}}{\left|\Omega_{u}\right|}$$

where Ω_*u*_ denotes the set of the *u*-th cluster, and |Ω_*u*_| denotes the number of elements in the set. Thus, the average FC series of the *s*-th subjects in the *u*-th cluster is obtained as


(6) $${\bar{h}}_{u}^{s}=\frac{\sum_{\left(i,j\right)\in \Omega_{u}}h_{i\,j}^{s}}{\left|\Omega_{u}\right|}$$

It is important to note that the size of *U* directly affects the difference of different clusters. In order to simulate the real high-order interaction of ROIs, we set the parameter *U* as one of {300, 400, 500, 600, 700, 800}. The cluster parameter *U* will be automatically searched in subsequent experiments, and the effect will be given in the discussion section. Based on the average FC series obtained in Eq. 6, the high-order functional connectivity between the *u*-th cluster and the *v*-th cluster can be expressed as $H_{u\,v}=\text{corr}\,\left({\bar{h}}_{u},{\bar{h}}_{v}\right)$. The HFCN can be defined as


(7) $$C_{D\,H}=\left[\begin{array}{c} H_{11}\,\,H_{12}\,\,\cdots \,\,H_{1\,U}\\ H_{21}\,\,H_{22}\,\,\cdots \,\,H_{2\,U}\\ \,\vdots \;\vdots \;\ddots \,\vdots \\ H_{U\,1}\,\,H_{U\,2}\,\cdots \,\,H_{U\,U} \end{array}\right]$$

where *C*_*DH*_ denotes the inter-modulation relationship between the functional connections of multiple ROIs, and reflects the spatial variability of the brain network. Furthermore, the HFCN obtained by “correlation’s correlation” strategy assumes that the functional brain network is fully connected. In order to better represent the real characteristics of the brain, we sparse the HFCN with percentage thresholding (edges with top λ_*HD*_% connection strength are retained) (Qiao et al., 2018). The high-order network thresholding parameter λ_*HD*_ range is {0:10:90}, which will be automatically searched in subsequent experiments, and the effect will be given in the discussion section.

### Feature extraction and selection

#### Temporal variability feature extraction

Temporal variability is a measure that describes the degree to which the FC of a particular ROI to all other ROIs changes over time (Zhang J. et al., 2016). The temporal variability of *i*-th ROI can be expressed as


(8) $$V_{i}=1-\bar{c\,o\,r\,r\,\left(w_{i}\,\left(k\right),w_{i}\,\left(l\right)\right)},k,l=1,2,3,\ldots ,K,k\neq l$$

where *w*_*i*_(*k*) = [*W*_11_(*k*),*W*_21_(*k*),⋯,*W*_*N*1_(*k*)]^T^ ∈ ℝ^*N*×1^ denotes FC under the *k*-th window between the *i*-th ROI and all other ROIs, *k* and *l* represent different time windows. The $\bar{c\,o\,r\,r\,\left(w_{i}\,\left(k\right),w_{i}\,\left(l\right)\right)}$ denotes the average of PC coefficient in different time windows for *i*-th ROI. The $\bar{c\,o\,r\,r\,\left(w_{i}\,\left(k\right),w_{i}\,\left(l\right)\right)}$ compares the functional architecture, i.e., overall functional connectivity profile associated with ROI *k* across different time windows. Then, a deduction from 1 indicates temporal variability of a ROI. In this way, it is possible to both target specific ROIs and assess the extent to which the functional architecture has changed over time at the global level.

#### High-order network topology feature extraction

We use the node degree and weighted-graph local clustering coefficient (Rubinov and Sporns, 2010) to extract features, separately. The node degree is the number of nodes directly connected to *i*-th node. The degree of the *i*-th node is defined as


(9) $$D_{i}=\sum_{j=1}^{n}W_{i\,j}$$

The weighted-graph local clustering coefficient quantifies the probability that neighbors of this vertex are also connected to each other, which can better characterize the clique structure of the FCN. The weighted-graph local clustering coefficient *f*_*i*_ for vertex *i* can be defined as follows


(10) $$t_{i}=\frac{1}{2}\,\sum_{j,h\in N}{\left(W_{i\,j}\,W_{i\,h}\,W_{j\,h}\right)}^{1/3}$$

(11) $$f_{i}=\frac{1}{n}\,\sum_{i\in N}\frac{2\,t_{i}}{r_{i}\,\left(r_{i}-1\right)}$$

where *j* and *h* are the neighbor nodes of node *i*, *r*_*i*_ is the number of neighbor nodes of node *i*, and *t*_*i*_ is the number of edges between all nodes connected to node *i*. The number of HFCN nodes depends on the number of clusters *U*, and the dimensions of the weighted-graph local clustering coefficient and node degree are both *U*.

#### Feature selection

The temporal variability extracted from DFCN is low-order features (*N* dimensional vectors), and the weighted-graph local clustering coefficient and node degree extracted from HFCN are high-order features (2*U* dimensional vectors). In order to remove irrelevant and redundant features for improving the generalization performance, Lasso regression algorithm (Tibshirani, 1996) is employed to find the most discriminative features relevant to SZ.

For considering the interaction among features, Lasso regression combines all features (with the dimension of *m* = *N* + 2*U*) to select feature subsets and the involved ℓ_*1*_-norm sparsity regularization is used to control the dimension of feature subsets. Lasso can be defined as


(12) $$\min_{\alpha }\frac{1}{2}\,{||Y-F\,\alpha ||}_{2}^{2}+\lambda_{F\,S}\,{||\alpha ||}_{1}$$

where *Y* = [*y*_1_,*y*_2_,….,*y*_*S*_] ∈ ℝ^*S*×1^ denotes the ground-truth label of the subjects (i.e., 1 for SZ, −1 for HC), and *F* ∈ ℝ^*S*×(*m* + 1)^ denotes a sparse dictionary that consists of the original feature matrix of *S* subjects and a column vector with all elements 1. α denotes the regression coefficient vector, and the position of its non-zero elements is the index of the selected feature in original feature set. λ_*FS*_ represents the penalty coefficient, which is used to control the sparsity of the feature subset. λ_*FS*_ is automatically searched in the experiments and its impact on model performance is given in the discussion section.

## Experiments and results

### Experiment settings

After selecting important features with Lasso, the support vector machine (SVM) with a linear kernel is trained for SZ identification. There are four hyperparameters in our proposed model, including window length *L* (range of {20:10:70}), clustering number *U* (range of {300:100:800}), high-order network thresholding λ_*HD*_ (range of {0:10:90}), and Lasso feature selection parameter λ_*FS*_ (range of {0.1:0.1:0.6}). The nested leave-one-out cross-validation (LOOCV) is used to evaluate classification performance and optimize those hyperparameters. Specifically, for *S* subjects in the dataset, *S*-1 subjects are used for training while the left-out one is used for testing. This procedure is repeated *S* times for evaluating the classification performance. To determine the optimal combination of the above four parameters, LOOCV is executed again on *S*-1 training subjects in the above process. Then, by applying the combination of optimal parameters on the *S*-1 different training subsets, we train *S*-1 classifiers to classify the test subject, and the final classification result is determined via majority voting. After repeating the above process *S* times, an overall cross-validation classification accuracy is calculated.

### Evaluation methodology and results

#### Evaluation metrics

The five metrics are employed to evaluate the performance, including accuracy (*ACC*), sensitivity (*SEN*), specificity (*SPE*), area under curve (*AUC*), and receiver operating characteristic (ROC). Those evaluation metrics can be defined as *ACC* = (*TP* + *TN*)/(*TP* + *FN* + *TN* + *FP*), *SEN* = *TP*/(*TP* + *FN*), *SPE* = *TN*/(*TN* + *FP*), where *TP*, *TN*, *FP*, and *FN* are true positive, true negative, false positive, and false negative, respectively.

#### Method comparison

**FIGURE 2:**
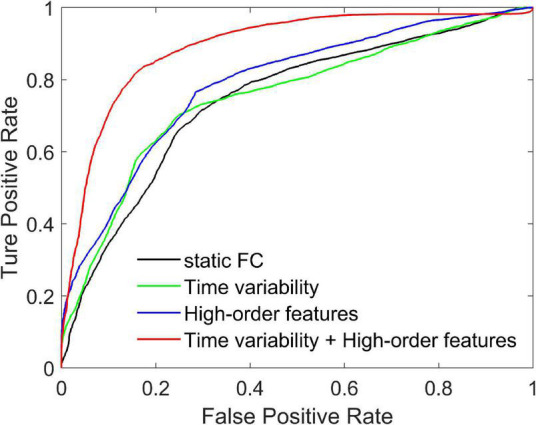
Receiver operating characteristic curves achieved by four different methods in SZ vs. HC classification.

We compare three methods with features extracted from SFCN, DFCN, and HFCN, respectively. The feature selection and classification procedures are same with our proposed method. Specifically, the upper triangular in static FC network (i.e., the matrix **W**) is extracted and converted into a long vector. The vectors of all subjects are standardized and used as input to SVM after feature selection. Similarly, the temporal variability features and high-order features extracted from the low-order DFCN and the HFCN are separately used as the input of SVM after feature selection. The classification results are listed in Table 2 and the ROC curve is shown in Figure 2.

**TABLE 2:** The comparison of performance in the classification of SZs and HCs by different methods.

Method	*ACC* (%)	*SEN* (%)	*SPE* (%)	*AUC* (%)
Static FC	68.60	77.19	60.94	74.42
Time variability	71.90	68.42	75.00	75.27
High-order features	73.55	75.44	71.88	78.84
Time variability + high-order features	81.82	82.46	81.25	89.26

It can be seen from Table 2 that the performance of the static FC method is less than 70%. This may be because the SFCN only delineates the functional connectivity between paired ROIs throughout the scan, ignoring the dynamic functional interactions between multiple brain regions during the scan. The temporal variability method is improved by 3.3% compared to the static FC method. This illustrates the DFCN and temporal variability method of simulating the dynamic interaction of the brain by measuring the short-term correlation of brain regions, which is beneficial to the capture of discriminative features. Through the clustering of FC series and the calculation of high-order correlations, HFCN can further characterize the modulation relationship between FC of multiple ROIs, thereby characterizing the complex abstract interactions of the brain. Therefore, the high-order features (node degree and graph-weighted local clustering coefficients) extracted from HFCN can further improve the classification performance. Finally, the classification performance can be effectively improved by fusing low- and high-order features. This may be because temporal variability and high-order topological features describe properties between brain regions from different perspectives, indicating that the complementary information is critical for disease diagnosis.

### Connectivity network analysis

**FIGURE 3:**
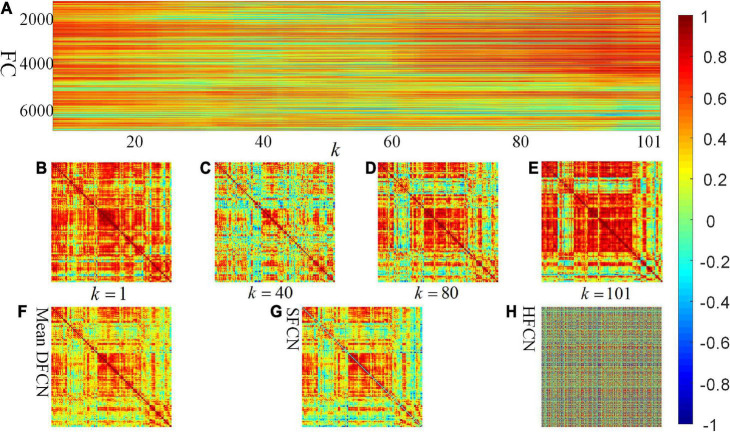
Visualization of SFCN, DFCN, and HFCN when the window length *L* = 40. **(A)** All the rearranged DFCN upper triangular elements along the time window, where the horizontal axis represents the 101 time windows, and the vertical axis represents the 7,140 functional connectivities. **(B–E)** The DFCN sub-networks under the *k*-th time windows, where *k* = 1, *k* = 40, *k* = 80, and *k* = 101, respectively. **(F,G)** The average of the sub-networks under whole time windows of DFCN and the static FC network based on pair-wise correlation of BOLD signals, respectively. **(H)** The constructed high-order FC network.

One participant is randomly selected to investigate the constructed connectivity network, shown in Figure 3. It can be seen from Figures 3F,G that there is less difference between the brain network constructed by SFCN using the full time series and the averaged DFCN. This may be because both are essentially correlation measures for BOLD signals, while subtle differences may be the short-term FC properties obtained by DFCN. From Figures 3A–E, it can be seen that measuring the correlation of BOLD signals at a fine-grained level can effectively reflect the dynamic functional interaction of ROIs. It is worth noting that the FC intensity varied significantly throughout the scanning phase. This change is reflected in spatially and temporally, that is, the FC has undergone interactive reorganization in different ROIs, which changes the modular structure of the brain network. Figure 3H shows that network topology in the HFCN is more complex, implying it containing more high-order ROI’s interaction.

**FIGURE 4:**
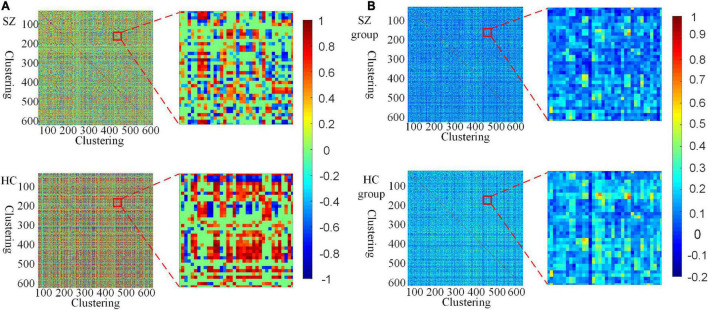
Visualization of HFCN for SZ and HC (*L* = 40, *U* = 600, λ*_*HD*_* = 0.5). **(A)** The HFCN and local details of the randomly selected SZ patient and HC. **(B)** The averaged HFCN and local details of SZ and HC groups.

Furthermore, we investigate the connectivity differences in the HFCN of SZ and HC from both individual and group perspectives. From Figures 4A,B, it can be seen that the high-order functional connectivity of HC is overall higher than that of SZ, which is consistent with the existing researches on lower-order brain networks (Su et al., 2013; Yu et al., 2020). This illustrates that there is a wide range of functional disconnections in SZ patients (Lynall et al., 2010). This disconnection is manifested in complex functional interactions across multiple ROIs (Li et al., 2019), leading to abnormalities in the functional separation and integration characteristics of SZ.

## Discussion

### Model parameter sensitivity analysis

To further analyze the influence of different parameters on the classification results, the classification performance under different window *L*, different cluster *U*, as well as different parameter combinations are discussed.

#### The effect of *L* and *U* on the performance

**FIGURE 5:**
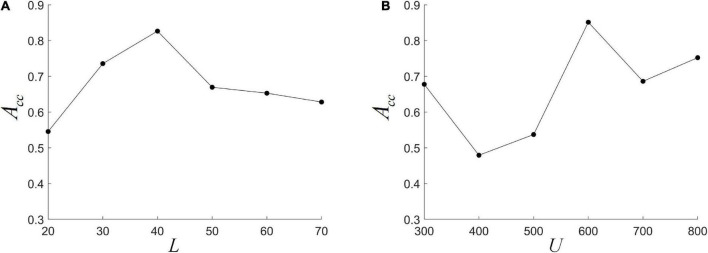
The effect of different parameters on classification performance. **(A)** The different window length *L*; **(B)** the different cluster number *U*.

We fix *L* at one of {20, 30, 40, 50, 60, 70}, and use nested LOOCV to optimize several other parameter combinations to analyze the effect of *L* on model performance. It can be seen from Figure 5A that *ACC* first increases and then decreases with *L*, and reaches the highest when *L* = 40. This indicates that the size of the *L* is important for the detection of dynamic interactions between ROIs. A smaller window length can capture shorter-term functional changes, but is more susceptible to noise. A larger window length would make the performance more stable, but the dynamic information cannot be effectively detected. This is consistent with previous research (Li Y. et al., 2020). In addition, when the *L* becomes large, the length of the FC series decreases, which may lead to a decrease in the reliability of HFCN. The choice of cluster number *U* also has a greater impact on the classification results. As shown in Figure 5B, the impact of *U* on performance is analyzed by fixing the number of clusters *U* to be one of {300, 400, 500, 600, 700, 800}. It can be seen that *ACC* is more sensitive to changes in the number of clusters, and the highest *ACC* can be obtained when *U* is 600.

**FIGURE 6:**
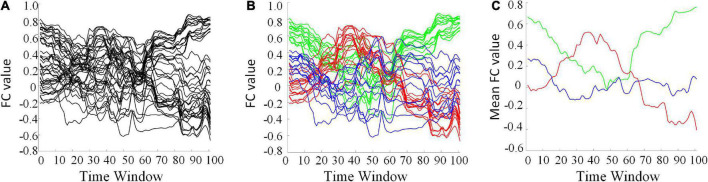
Some selected FC series and their clustering results (*L* = 40, *U* = 600). **(A)** The original FC series. **(B)** Red, green, and blue indicate three different clusters. **(C)** The averaged FC series of the three clusters, separately.

The clustering algorithm can reduce the dimension of HFCN and effectively decrease the computational complexity as well as the redundant features. The FC series of some ROI pairs are depicted in Figure 6A. For many ROI pairs, their temporal correlations are significantly different, such as the FC strength and change trend. From Figures 6B,C, it can be seen that the functional connectivity order of three clusters has a consistent change trend within the cluster, but has a significantly different change trend among the clusters. The clustering algorithm is able to find the underlying dominant dynamic patterns in all FC sequences.

#### The effect of different parameter combinations on the performance

**FIGURE 7:**
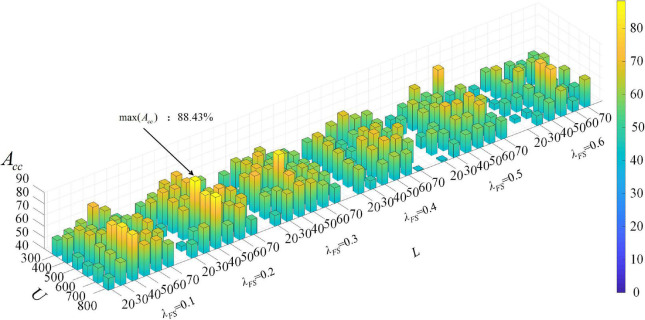
Statistical classification accuracy is estimated by our proposed method with different values of hyperparameters. For clear visualization, HFCN thresholding parameter λ_*HD*_ is set to 0.5. These results are obtained by LOOCV on whole data.

To further analyze the impact of different parameter combinations on the performance, we conduct an additional LOOCV experiment, and the accuracy under different combinations of parameters (*L*, *U*, λ_*FS*_) with HFCN threshold parameter λ_*HD*_ = 0.5 is shown in Figure 7. It can be seen that when *L* = 40 and *U* = 600, the accuracy is generally higher, which is consistent with the previous results when *L* and *U* are analyzed separately. In addition, the Lasso feature selection parameter λ_*FS*_ has a greater impact on the classification results. When λ_*FS*_ is set to a small value (0.1–0.3), high classification performance can be obtained. This is because λ_*FS*_ controls the number of features in the feature selection process. When λ_*FS*_ is too large, some discriminative features are discarded together with redundant features, which may be detrimental to the classification performance.

### Most discriminative features

**FIGURE 8:**
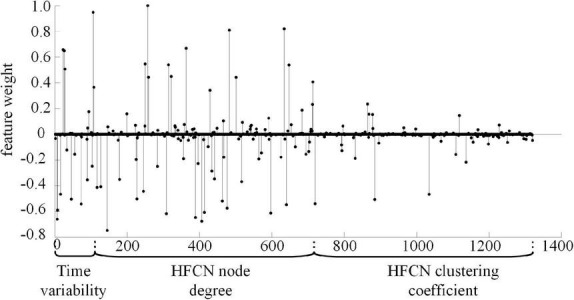
The normalized classification weights of all features (*U* = 600).

To find out the discriminative features or clusters for SZ identification, we fix the number of clusters *U* at 600 and analyze the importance of each feature in the classification. Specifically, during the feature selection process in each validation step, the selected features for classification might be different for different training datasets. We count the total weight of each feature in all training and classification, and use it to measure the classification contribution of features, as shown in Figure 8. It can be seen that only a few features have a large weight, indicating that they are the discriminative features for classification. Furthermore, most of the features with large weight belong to the time variability features and node degree features of high-order network, which reveals that these two kinds of features play a more important role in SZ diagnosis.

**FIGURE 9:**
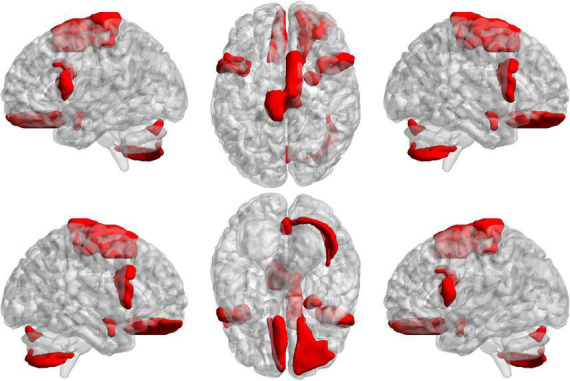
The projections of ROIs corresponding to the ten time variability features with the large weights on the cortical surface.

To further find the most important classification features, we conduct a separate analysis of time variability features and high-order features. For the time variability features, we take the absolute value of the normalized feature weights and sort them. Table 3 lists the brain regions corresponding to the top ten features with large weights. We can see that the brain regions with highest contribution including the *frontal lobes*, *Cerebellum7bR*, *Vermis7*, and *limbic system*, which are visualized in Figure 9. These regions have been suggested to be related to SZ by previous studies (Weinberger et al., 1994; Grace, 2000; Ichimiya et al., 2001).

**TABLE 3:** Brain regions corresponding to the 10 time variability features with large weights.

ROI index	ROI name	Anatomical zonation
106	Cerebelum7bR	Cerebellum
23	Gyrus rectus	Frontal lobe
7	Inferior frontal gyrus, opercular part	Frontal lobe
26	Medial orbital gyrus	Frontal lobe
8	Inferior frontal gyrus, opercular part	Frontal lobe
73	Paracentral lobule	Frontal lobe
28	Anterior orbital gyrus	Frontal lobe
46	Amygdala	Limbic system
16	Supplementary motor area	Frontal lobe
117	Vermis7	Cerebellum

**FIGURE 10:**
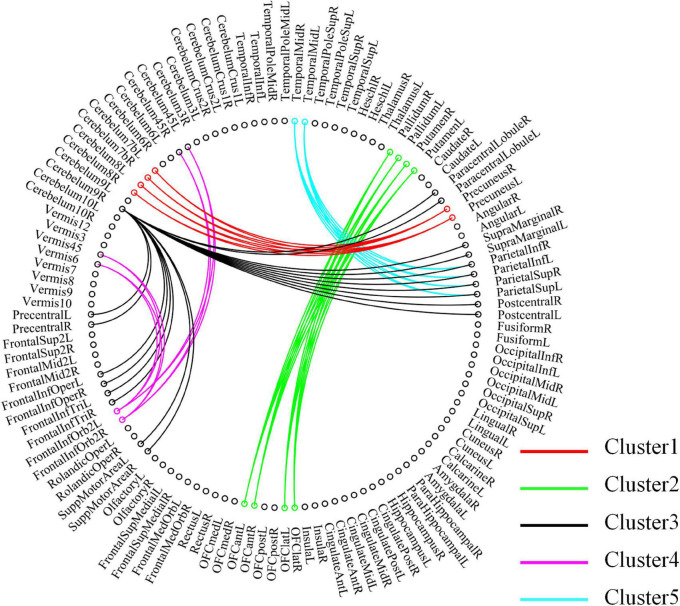
Functional connectivity contained in the five clusters with the largest classification weights. Each node in the figure represents a ROI, and the line between the two nodes represents functional connectivity. The functional connections contained in different clusters are shown in different colors, and the importance increases sequentially from Cluster 5 to Cluster 1.

**FIGURE 11:**
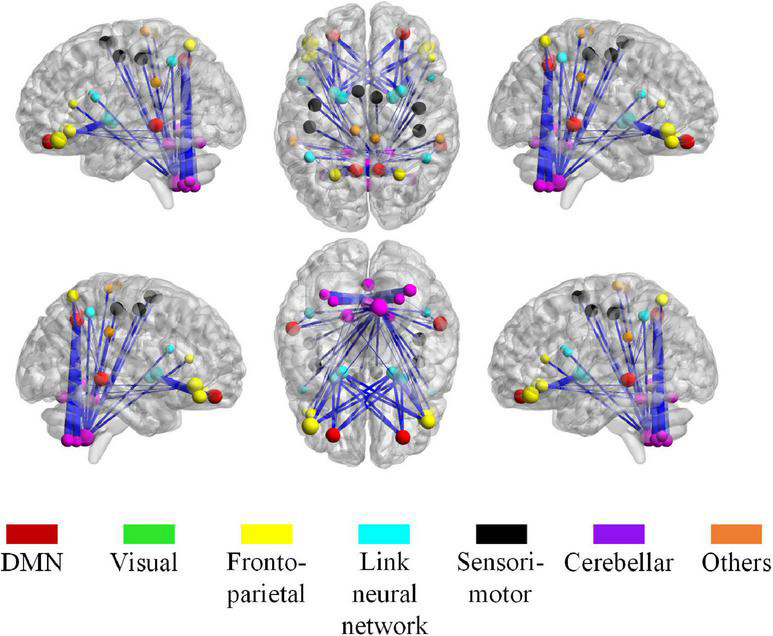
The most discriminative connections and brain regions in the whole brain view. The size of a node, calculated from the total weight of all clusters that containing the node, indicates the importance of the node during the classification. The thickness of the edge represents the classification weight of the cluster that connects the edge.

**FIGURE 12:**
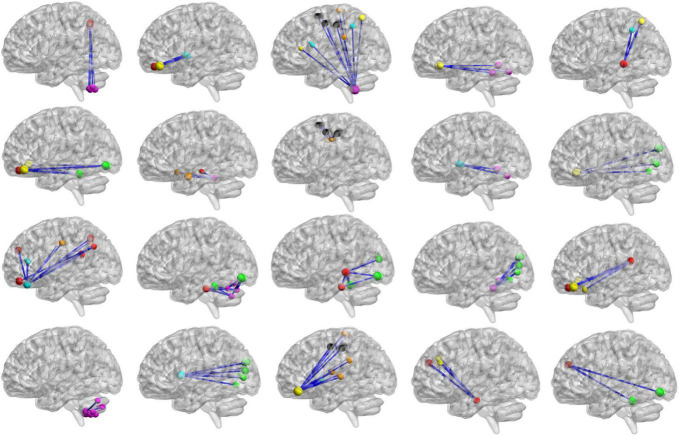
The top 20 clusters with the highest classification weight. The functional subnetworks represented by different colors are the same as in Figure 11.

For high-order features, we add the feature weights of the node degree and the weighted-graph local clustering coefficient of HFCN in classification to obtain the importance of a certain cluster. The five most important clusters are filtered out for classification and presented in the form of a chord diagram, as shown in Figure 10. It can be seen that the five clusters with the largest weights involve 58 functional connections and 21 brain regions (20 mirrored brain regions of both hemispheres among them). Five brain regions are consistent with Figure 9 and Table 3, including the *insular inferior frontal gyrus*, *paracentral lobule*, *retroorbital gyrus*, *supplementary motor area*, and *cerebellum7b*. Figure 11 shows the related FC within the five clusters. It can be seen that these functional connections mainly involve the *frontal lobe*, *parietal lobe*, and *cerebellum*. Among them, the frontal lobe-cerebellar and the parietal lobe-cerebellar connection are more numerous and weighted. Figure 12 shows the specific top twenty clusters with the highest classification weights. It can be seen that the *cerebellum* and *frontal lobes* still have constant importance in these top clusters. In addition, the visual network regions of the *occipital lobe*, such as the fusiform gyrus and the cortex around the talar fissure are also of greater importance for the diagnosis of SZ.

In this article, cerebellum (especially *Cerebelum7bR*) and *frontal lobe*-related features play the most important roles in SZ classification, which confirmed by features extracted from both DFCN and HFCN. In fact, abnormalities in the cerebellum and *frontal lobe* of patients with SZ have been identified in other studies (van den Heuvel et al., 2010; Wang et al., 2014). For example, Andreasen et al. (1996) found that SZ patients showed dysfunction of the prefrontal-thalamic-cerebellar circuit, and proposed the Cognitive Dysmetria hypothesis (Andreasen et al., 1998), which believed that abnormalities in the neural circuits connecting the thalamus and cerebellum may lead to abnormalities in cognitive control, coordination and affective aspects. This may be the underlying psychopathological cause of the complex symptoms of SZ. Functionally, the *frontal lobe* is involved in attention regulation, abstract rules, social behavior, etc., and is also linked to the symptoms of SZ. In terms of the cerebellum, it is not only mainly responsible for the function of motor control but also has been reported in the research of SZ that there are abnormalities of neural circuits between it and other cortex (Cao and Cannon, 2019; Kelly et al., 2021). In addition, the striatum and multiple regions of the parietal-premotor cortex (*paracentral lobule*, *postcentral gyrus*, *precuneus*, *supramarginal gyrus*, *inferior parietal gyrus*, and *superior parietal gyrus*) also show important roles in SZ classification (Salvador et al., 2005). The striatum has been a key region in the study of SZ, and has an important relationship with the dopamine hypothesis of the etiology of SZ (Li A. et al., 2020). Many antipsychotic drugs rely on blockade of dopamine receptors in the striatum (Kegeles et al., 2010). In the AAL2 template, the striatum is divided into three brain regions: *caudate nucleus*, *putamen*, and *pallidum*, two of which appear in the five clusters with the largest classification weight and show abnormal connectivity to the *orbitofrontal cortex*. The *paracentral lobules*, *postcentral gyrus*, and other brain regions of the parietal-premotor cortex are mainly involved in sensory-motor, spatial attention, etc. Abnormalities in these brain areas may explain the sensory, thinking and behavioral disorders in SZ.

## Conclusion

In this article, we propose a temporal-spatial dynamic functional connectivity analysis method that combines DFCN and HFCN for SZ diagnosis. Specifically, we first construct a DFCN and extract temporal variability features that reflect temporal dynamic information. Based on DFCN, the HFCN is constructed by means of clustering algorithm and “correlation’s correlation” strategy, and the node degree and graph-weighted local clustering coefficient features of the sparse HFCN are extracted. Then, the most discriminatory features are selected with Lasso method for SZ classification. The proposed method is verified on a real SZ dataset and demonstrates promising performance via comparison with the competitive brain network analysis methods. The abnormal brain regions detected in this paper can provide a direction for more detailed research on the pathology of SZ and the search for biological markers in the future.

## Data availability statement

The datasets presented in this study can be found in online repositories. The names of the repository can be found in the article.

## Ethics statement

The studies involving human participants were reviewed and approved by IORG0000256 – University of New Mexico. Written informed consent was obtained from all participants for their participation in this study.

## Author contributions

CP, HY, and RY have developed the modeling algorithm and architecture. XF and XZ have preprocessed the data. All authors have contributed to preparation of the article, figures, and tables, approved it for publication, and listed have made a substantial contribution to the work.

## Footnotes

1
http://fcon_1000.projects.nitrc.org/indi/retro/cobre.html
